# Coughs and Their Treatment

**Published:** 1903-02

**Authors:** 


### Coughs and Their Treatment.

Under the above title Drs. Alex. de Soto and C. W Crimpton, physicians to the Wayside Mission Hospital, Seattle, Wash., present some important observations upon this subject, which possess special importance in the winter season. Among other things they say: cough is a symptom, varying in intensity and character according to its cause, nor is the cause always situated within the respiratory organs themselves. It is essentially a reflex act depending upon an irritation of the respiratory center.

These sources of irritation may be subdivided as follows: dropping of mucus from the posterior nares in chronic catarrh; polypi, enlarged uvula or tonsils, defective closure of the glottis, irritations within the larynx from whatsoever cause, malignant or otherwise; bronchitis, pneumonia and pleurisy; gastric, when due to derangements of the stomach; cardiac disease, irritations of auditory canal and organic diseases within the abdominal cavity.

From the foregoing causes, it may be readily estimated that to arrive at the exact nature of the cause in any given case may not always be an easy matter. Nevertheless, we must relieve the patient, without risk of disturbing either digestive or circulatory systems. Any remedy which will attain this object in a goodly number of cases is of inestimable value to patient and physician.

Not until our attention was called to glyco-heroin (Smith) did we become acquainted with a remedy which we have used with almost unvarying success in coughs of every description, and in patients of all ages and conditions, without the slightest unfavorable effect.

The points which recommend this remedy may be briefly stated as follows: (1) palatability; (2) economy; (3 to 4 oz. being ample for a cure of the average case); (3) its immediate action, soothing the most trying cases; (4) its absolute freedom from unpleasant or unfavorable effects; (5) it is not only a palliative but a curative agent; (6) the hyoscyamus it contains reaches those trying cases of dry cough due to other causes than simple catarrhal irritation of the respiratory tract.

These observers declare that its action is almost specific and that it will give satisfaction in every case where results may be reasonably expected, and in many instances its beneficial effects go beyond the most sanguine expectations.

They report a number of cases in detail, treated in the hospital and in the out-patient department, from which the foregoing conclusions have been reached.

